# Experiences of Nature Through Immersive Virtual Reality Among People with Type 2 Diabetes Mellitus

**DOI:** 10.3390/ijerph23050615

**Published:** 2026-05-06

**Authors:** Monika Norberg, Elisabet Bohlin, Ann Dolling, Benno Krachler, Julia Elfving, Martin Gärdemalm, Kristina Lämås

**Affiliations:** 1 Department of Nursing, Umea University, SE 901 87 Umea, Sweden; monika.norberg@umu.se; 2 Department of Forest Ecology and Management, Swedish University of Agricultural Sciences, SE 901 83 Umeå, Sweden; elisabet.bohlin@slu.se (E.B.); ann.dolling@slu.se (A.D.); martin.gardemalm@slu.se (M.G.); 3 Department of Epidemiology and Global Health, Umea University, SE 901 87 Umea, Sweden; 4 Livsstilsmedicin Österåsen, Österås 306, SE 881 91 Sollefteå, Sweden; julia.elfving@rvn.se

**Keywords:** type 2 diabetes mellitus, nature assisted therapy, nature-based intervention, forest bathing, nature exposure, stress reduction, emotional wellbeing, attention restoration theory, immersive virtual reality, qualitative method

## Abstract

**Highlights:**

**Public health relevance—How does this work relate to a public health issue?**

- Type 2 diabetes (T2D) is a growing global public health challenge associated with long-term complications and increased mortality.
- Psychological distress and reduced wellbeing can hinder the maintenance of lifestyle-related behavioral change in T2D.

**Public health significance—Why is this work of significance to public health?**

- Lifestyle interventions are central in T2D management, yet sustaining behavioral change is often challenging.
- The study explores experiences of immersive virtual reality (IVR)–based nature exposure as a supportive approach for wellbeing in people with type 2 diabetes.

**Public health implications—What are the key implications or messages for practitioners, policy makers, and/or researchers in public health?**

- IVR-based nature experiences were perceived as calming and supportive of emotional wellbeing.
- Familiarity and personal meaning of the environments appeared central to the perceived restorative experience.
- IVR may complement, but not replace, real nature exposure, and could be particularly relevant for individuals with limited access to natural environments.

**Abstract:**

This study explores experiences of spending time in immersive virtual reality with a natural environment among individuals with type 2 diabetes, aiming to enhance perceived wellbeing and reduce perceived stress. Seventeen participants with type 2 diabetes took part in a multimodal lifestyle education program and used immersive virtual reality with natural environment over a six-month period, selecting from a number of 30 min serene natural environments. Semi-structured interviews were conducted and analyzed using qualitative content analysis. Participants described immersive virtual reality with a natural environment experience as providing tranquility, inspiration, and a sense of transcending time and space (A). Feelings of calm and mental withdrawal from everyday demands were often reported (A2), and memories were evoked (A2). Some participants experienced these effects as extending beyond the immersive virtual reality with natural environment sessions themselves (A3–4). At the same time, several factors were identified that could disrupt the restorative experience (A5), including technical issues, individual preferences for specific environments, health- or situation-related constraints, and difficulties establishing a regular routine for headset use. Immersive virtual reality with natural environment was generally viewed as a valuable complement to real-world nature experiences, particularly for individuals with limited access to outdoor environments (B1–2). Overall, the findings suggest that immersive virtual reality with natural environment experiences may offer a supportive resource for enhancing emotional wellbeing and managing stress in people with type 2 diabetes, while not replacing the benefits of actual nature exposure.

## 1. Introduction

Type 2 diabetes (T2D) is a chronic condition associated with serious complications and elevated mortality [1]. Long-term outcomes depend heavily on sustained lifestyle changes such as healthy eating, regular physical activity, weight management, and stress regulation [2]. Despite good evidence for the effectiveness of structured diabetes education, maintaining such behavioral changes over time remains difficult [3].

A major barrier is the psychological burden of living with T2D. Many patients experience diabetes-related distress, reduced motivation, and impaired quality of life [1,4]. Stress and anxiety can also directly worsen glycemic control through neuroendocrine and inflammatory pathways [5]. This highlights the need for supportive interventions that reduce stress, increase wellbeing, and thereby strengthen motivation.

### 1.1. Nature Exposure as a Supportive Health Intervention

Research indicates that natural environments can reduce stress, enhance mood, and support psychological recovery [6,7,8,9,10,11,12,13,14,15]. Short forest exposures have shown psychological and physiological benefits in exhaustion disorder [16,17,18]. Green space access has been linked to lower T2D prevalence and fasting glucose [19]. There is also tentative evidence that nature contact may support motivation for lifestyle change [20] and improve affective responses during exercise, with possible implications for adherence—though findings remain inconsistent [21]. Together, these findings suggest that exposure to nature addresses key psychological mechanisms—stress, low motivation, cognitive fatigue—that often undermine sustained lifestyle change in T2D.

### 1.2. Immersive Virtual Reality as an Accessible Form of Nature Exposure

Immersive virtual reality with natural environment (IVR-N) enables individuals to experience restorative natural environments even when access to real nature is restricted. Interest in immersive virtual reality (VR) as a method for delivering nature-based interventions is increasing. VR-based representations of natural environments are being increasingly utilized in healthcare and rehabilitation contexts, including interventions for individuals with cancer, stress-related conditions, and limited mobility [22,23,24]. IVR-N can elicit affective and relaxing responses similar to those of real environments [25], and perceived realism appears crucial for these benefits [26].

Recent systematic reviews, e.g., refs. [27,28,29] suggest that IVR-N generally reduces stress and may enhance wellbeing, though heterogeneity among studies is substantial. Some nature scenes may evoke fear in certain individuals, and a sense of safety appears to mediate positive effects [28], underscoring the value of exploring user preferences and experiences. Such interventions have demonstrated measurable stress-reducing effects based on physiological indicators [30], suggesting that virtual exposure may serve as a viable complement to physical nature contact.

Evidence for IVR-N in T2D is scarce. One study found that an IVR-supported exercise program improved blood glucose levels and muscle mass [31], but no studies to our knowledge have examined IVR-N as psychological support in this population.

### 1.3. Need for Qualitative Research on IVR-N Experiences in T2D

Qualitative research on IVR-N for health promotion is limited. In a meta-synthesis of older adults’ experiences of IVR, the environments are not clearly described but seem to include more than the natural environment [32]. They found initial hesitation but generally positive reactions among older adults after use, with IVR enabling participation in otherwise inaccessible environments [32]. Virtual-nature exposure also improved attention and mood among community-dwelling older adults [33]. In palliative care, including every type of IVR-therapy, IVR was well-suited for some but caused discomfort for others, highlighting the importance of exploring acceptability in specific groups [34]. Given the psychological challenges in T2D and the limited evidence on IVR-N in this population, a clear knowledge gap exists.

### 1.4. Aim

The aim of this qualitative study was to explore the experiences of using IVR-N as a potential therapeutic support to enhance perceived wellbeing and reduce perceived stress among people with T2D.

## 2. Method

### 2.1. Design

This study is part of the research program Natureach—Nature Reachable for everyone to improve health and wellbeing (https://www.slu.se/Natureach, 2 May 2026). The purpose of the program is to develop and evaluate virtual nature experiences for clients and healthcare professionals and is funded by Interreg Aurora.

Our study has a qualitative design with an inductive approach to exploring experiences of using a VR headset (Pico 4 Enterprise, Pico Interactive, Beijing, China) to facilitate nature experiences. The study has been conducted in northern Sweden during 2023–2025. The data was collected via interviews and analyzed with qualitative content analysis.

### 2.2. Setting

The participants were all attending a multimodal lifestyle education program at Lifestyle at Österåsen, Västernorrland, Sweden. The aim of the overall program is to improve diet and physical activity, as well as reduce stress. In this study, we focused specifically on the stress management component. What is novel is the use of VR-based nature as one component within the broader lifestyle intervention program. The study represents an attempt to identify an approach to stress management that may be appropriate for a subset of individuals, since not everything suits everyone. The education program includes one introduction week and three education weeks at Österåsen with a variety of treatments aiming at changing lifestyles that cause ill-health. After the introduction and treatments, the participants went home for six months and then returned to Österåsen for one follow-up week.

### 2.3. Study Sample

The inclusion criteria were to have T2D, be able to understand and speak Swedish, handle a VR headset, and attend a multimodal lifestyle education program at Lifestyle Medicine at Österåsen. During the recruitment period, January 2024–January 2025, 255 persons took part in the program; of those, 53 persons met the criteria. They were invited to an information session about the research project held at the start of each education program. Twenty-four patients provided written informed consent to participate in the study; however, five of them withdrew before leaving Österåsen. In total, 19 participants were recruited (seven men and twelve women), aged 43–74 years (mean age 60 years). Two of these 19 patients who brought headset devices home chose not to continue the intervention as they realized that they would not have sufficient opportunities to use headsets as intended. Reasons included lack of time and poor mental health at the beginning of the period at home, which led to a decrease in motivation for participation. These two participants were therefore not included in the interview study. The lifestyle education program is based on lifestyle modifications at the individual level. As the aim of the study was to assess perceived changes within individuals over time, no control group was included.

### 2.4. Intervention

In addition to the ordinary education program at Österåsen, the participants received a nature-based intervention three times/week during the three-week health course. The intervention aimed to promote mindful presence in nature, through spending time at a self-selected spot in the forest at Österåsen and during the home period, through immersive virtual representations of natural environments. During the period at Österåsen, the participants were introduced to the VR headset and had the opportunity to test and become familiar with the equipment before going home. After the three-week visits at Österåsen, the participants were instructed to use the VR headset at home for 30 min/three times per week for six months before the ordinary follow-up week at Österåsen. The evidence regarding the therapeutic dose of the intervention is limited; therefore, a pragmatic approach was adopted.

The participants were able to choose from the different natural environments presented through the VR headset. The VR headset included a virtual library in which participants could choose the environment they wished to spend time in at the beginning of each session. All environments were recorded in northern Sweden and Finland and were thus presumed to be familiar to the participants. The locations were selected to represent a diverse range of natural landscapes (forest, lake, sea, river, mire, meadow), which have been found to improve mood and restoration [35], as well as variation in season, weather conditions, and time of day. This diversity aimed to accommodate individual preferences while maximizing the environment’s restorative potential according to previous research [32].

The choice of environments for filming was based on the theories Attention Restoration Theory (ART) and Stress Reduction Theory (SRT). Both theories emphasize the psychological relationship between humans and nature, emphasizing nature’s potential to support mental wellbeing. Nature’s capacity to influence wellbeing has been hypothesized to be partly rooted in evolutionary adaptations to natural environments. ART, developed by Kaplan and Kaplan [36], focuses on the cognitive benefits of nature. The theory posits that direct attention capacity is essential for information processing in everyday life. This process, though, requires considerable mental effort, which can lead to direct attention fatigue. Nature is believed to have a restorative effect on direct attention by facilitating soft fascination. Soft fascination is described as an effortless attention that provides the opportunity to observe and enjoy nature while allowing thoughts to wander and giving space for reflection. SRT, proposed by Ulrich et al. [37], emphasizes nature’s affective benefits in reducing psychological and physiological stress. The theory suggests that exposure to nature elicits affective and physiological responses that promote stress recovery. This stress recovery is explained by reduced sympathetic activity and increased parasympathetic activity. Stress reduction, in turn, affects cognitive ability [38].

The scenes were filmed using 360° cameras positioned in tranquil, scenic locations, generally aiming for an open view with as few distracting elements as possible and with natural shelter in the opposite direction. The choice of the specific spot at each location was made to simulate the experience of sitting comfortably in a peaceful natural setting, allowing participants to freely explore the surroundings through head movements and soft fascination. To enhance the sense of presence and support a positive sensory experience, natural soundscapes were made, utilizing both on-site recordings and different site-appropriate nature sounds. Some environments also featured the presence of animals. Furthermore, the choice of environments was based on interviews with researchers who had previously conducted studies at Österåsen.

### 2.5. Data Collection

When the participants returned to the follow-up week, they were invited to take part in an interview. Of nineteen participants, two had not used the VR headset and did not participate in the interviews. Seventeen interviews were conducted; fourteen of these interviews were performed face-to-face in a room at Österåsen, and three interviews were performed online in an e-meeting room. The interviews were conducted by two of the authors, both of whom have experience in conducting research interviews within a nursing context. The interviews were semi-structured, and an interview guide with topics of interest was used; all authors were involved in developing the interview guide. The topics addressed the participants’ experiences and emotions during and after spending time in the natural environment in IVR-N, preferences regarding choice of natural environment, usability and management of the technical device, and perceived effects on motivation to spend time in real nature. The questions were open-ended, for example: Can you tell me about your experience of being in the virtual environment? Also, follow-up questions were used, for example: Can you tell me more about…? The interviews were tape-recorded and lasted between 17 and 46 min.

### 2.6. Data Analysis

The interviews were transcribed verbatim and read several times. The analysis followed the steps described in Graneheim and Lundman [39] and included dividing data into meaning units, condensing the meaning units, formulating codes, sorting codes into subcategories and categories, subthemes, and themes. The sorting and formulation of codes, subcategories, categories, subthemes, and themes have been discussed and critically reflected upon by the authors. The analysis was conducted iteratively, moving between meaning units, subcategories and categories, subthemes and themes to ensure trustworthiness.

### 2.7. Ethical Approval

The participants were informed both orally and in writing regarding the study and gave their informed consent to participate. They were assured confidentiality in the presentation of the study. Prior to the interview, participants were informed that they could withdraw from the interview at any time without providing a reason. The study has been approved by the Swedish Ethical Review Authority Dnr 2020-01701 and 2024-00694-02.

## 3. Results

The participants’ narratives of their experience of IVR-N were formulated in two themes and seven subthemes (Table 1). In the following text, interview quotations are written in italics. 

### 3.1. A. Finding Tranquility and Inspiration While Transcending Time and Space

This theme captures participants’ experiences of IVR-N to temporarily disengage from their immediate surroundings and enter a tranquil experiential space. The use of the VR headset enabled a sense of being away from everyday demands, which was interpreted as a form of spatial transcendence. In parallel, participants described how virtual nature evoked memories of earlier life experiences, creating an interplay between past and present that shaped their perception of the IVR-N. The tranquility experienced during IVR-N sessions was often described as extending beyond the immediate exposure, manifesting as sustained calmness, improved focus, and, for some, better sleep. In addition, IVR-N experiences appeared to inspire renewed interest in engaging with real natural environments and fostered a more mindful and attentive way of being in nature. At the same time, participants described factors that could hinder the experience of tranquility. These included technical limitations of the IVR equipment, individual preferences regarding the virtual environments, and health-related or situational constraints that made it difficult to establish regular use. The theme comprises five subthemes: Feeling a peaceful retreat from the demands of everyday life (A1), Being connected to the past and the present (A2), Feeling a lingering sense of harmony and presence (A3), Being inspired to engage with nature in new ways (A4), and Being hampered to find tranquility (A5) (Table 1).

#### 3.1.1. A1 Feeling a Peaceful Retreat from the Demands of Everyday Life

Participants described IVR-N as offering a temporary retreat from everyday demands. Being immersed in the virtual environments enabled a sense of stepping out of ordinary time and space, often described as being “*in one’s own bubble*”. This experience was associated with calmness, relaxation, and a sense of safety, allowing participants to slow down and be present with their thoughts. Easy access to nature and the possibility of engaging with natural environments without physical effort or perceived risks further facilitated these tranquil experiences. One participant explained: “*You can take a break, it’s nice to be in your own bubble. Really nice, especially when you’re stressed—you’re forced to slow down.*” Another participant described using the headset during lunchtime at work: “*I started using them at the office during lunchtime … I sat down on the chair next to my desk. It was nice, I could disconnect from work during that moment, rest my head from the numbers, just be.*”

#### 3.1.2. A2. Being Connected to the Past and the Present

Spending time in IVR-N evoked an intertwined experience of past and present. The virtual environments appeared to trigger memories of earlier life experiences, which in turn shaped how the IVR-N was perceived. This temporal interconnection was interpreted as a form of transcending time. Participants recalled past activities in nature, such as places they had lived, summer swims at beaches, or campfires and barbecues shared with friends. These memories were often described as warm and meaningful, but could also carry elements of longing, loss, or reminders of physical limitations. One participant reflected: “*It reminded me of the cabin where I wanted to be at—I miss it. It reminded me of my former life. Before the accident… The calm. The dream… to sit there, physically.*” Memories did not only arise from the IVR-N experience but also appeared to enrich it. Familiar sensory experiences—such as the warmth of the sun, the smell of the forest, or the sensation of sitting by a fire—were imaginatively recalled and integrated into the virtual experience. As one participant expressed: “*I felt the sun because I know how the sun feels. I have it in my mind, like a sensation. I felt the forest—I know how it smells, even though it wasn’t like that in the film.*” Previous emotional experiences of real nature also influenced which environments were perceived as most meaningful. Participants described preferring environments that evoked positive personal memories, such as boat trips with a parent, picnics, fishing trips, or sitting around campfires. As one participant noted: “*Those environments that reminded me of nice places I’ve been, I liked them most*.”

#### 3.1.3. A3 Feeling a Lingering Sense of Harmony and Presence

Participants described the effects of IVR-N that extended beyond the immediate session. Feelings of calmness, tranquility, joy, and ease in both body and mind were often described as lingering afterwards. When using the headset in the evening, some participants reported that it became easier to fall asleep and that stress and anxiety seemed to decrease, allowing a shift into a different emotional state. One participant explained: “*There has been a big difference when I’ve put them on and used this. Even when I was very anxious, I was able to do it anyway. And it has felt better*.” Another participant added: “*Afterwards I have felt a bit different compared to before. I’ve felt more at ease. It lingers. You’ve broken that stress*.”

Participants also described an increased sense of presence and improved ability to focus. Wearing the IVR headset reduced external distractions, such as mobile phones, and facilitated mental clarity. Some participants deliberately used IVR-N during lunch breaks to sustain concentration during the afternoon: “*I’ve had a busy morning at work, then I used the headset. And in the afternoon, I felt that I had a greater focus on what I was doing.*”

#### 3.1.4. A4 Being Inspired to Engage with Nature in New Ways

Experiences of IVR-N appeared to inspire participants to engage with real outdoor environments in new or renewed ways. Several described a desire to spend more time outdoors, explore new places, or reconnect with nature socially. One participant described organizing an outdoor gathering with friends after experiencing a campfire scene in IVR-N, while another reflected: “*I can imagine taking a walk by the sea more often. It felt cool there in the* IVR-N. *Good to discover new places—otherwise you just go where you usually go*.” The IVR-N experiences also seemed to influence participants’ mindsets when being outdoors, encouraging a more mindful and attentive way of engaging with nature. Participants described becoming more present and intentionally disconnecting from digital devices when outside: “*Now I look at nature in a different way when I’m outside. I have a little more mindful thinking—that I am here and now.*” Some participants expressed a growing longing to be in nature, even if the motivation for physical exertion was less prominent than before, emphasizing instead the value of simply being in natural environments.

#### 3.1.5. A5 Being Hampered to Find Tranquility

Despite many positive experiences, participants also described factors that hindered their ability to achieve tranquility through IVR-N. Technical shortcomings were one such factor, including rapid battery depletion, difficulties starting sessions, and challenges regulating sound. Some participants experienced the headset as uncomfortable due to its weight or the requirement to remain seated: “*I started to feel pain (when I used the headset) because I was wearing my glasses. I couldn’t see without them, and after a few minutes I felt the pressure, and then I almost got claustrophobic instead.*” Individual preferences also influenced whether IVR-N was experienced as calming or stressful. The same environment could be perceived as soothing by one participant and irritating by another. Preferences varied regarding movement, level of activity, animals, and soundscapes. Some participants appreciated dynamic elements such as water movement or animals, while others preferred more static and quieter environments. Preferences also appeared to change over time and in relation to health status. One participant reflected: “*Now I can almost not understand how I could choose the sea, that roaring one. Maybe I wasn’t really feeling well back then*.” Finally, some participants described difficulties in establishing IVR-N use as a meaningful routine in everyday life. Competing priorities, lack of energy due to physical or mental health, or perceiving the environment as monotonous reduced motivation. As one participant expressed: “*Now when I’ve been burnt out or exhausted, I’ve had a hard time motivating myself to do things, including using the VR headset. Which is a great shame.*”

### 3.2. B Perceiving Nature in IVR as a Surrogate for Real Nature

This theme reflects participants’ comparative reflections on experiences of IVR-N and in real outdoor environments. Participants articulated two interrelated but contrasting perspectives. On the one hand, IVR-N was perceived as realistic and, in some situations, comparable to real nature. On the other hand, participants emphasized that real nature offers a multisensory and embodied experience that IVR-N cannot fully reproduce. Consequently, IVR-N was not regarded as a replacement for authentic natural environments, but rather as a surrogate that could partially fulfill the functions of real nature when access was limited.

#### 3.2.1. B1 Perceiving Nature in IVR as Comparable to Real Nature

Nature experienced through IVR-N was described as realistic and, at times, closely resembling real nature. Participants expressed that sitting in a forest or by a fire in IVR-N could feel similar to being in the corresponding real-world environment. The immersive quality of the technology was highlighted as a key factor mediating this sense of realism. Earlier experiences of real nature also appeared to enhance the perceived realism of IVR-N. Familiarity with natural settings, including remembered sensory impressions such as warmth, smell, or sound, contributed to the feeling of “being there”. As one participant described:

“*It felt like I was sitting there. It felt like I could even feel the heat of the fire*.”

For some participants, this sense of realism made IVR-N a meaningful alternative when real nature was temporarily inaccessible.

#### 3.2.2. B2 Perceiving Nature in IVR-N as a Complement but Not a Replacement for Real Nature

At the same time, participants emphasized that IVR-N could not fully replace real nature. A central distinction concerned the absence of multisensory engagement. Participants described how sensations such as the warmth of the sun, wind against the skin, and natural scents contributed to a deeper sense of connectedness in real outdoor environments—an experience they felt was only partially achievable in IVR-N. One participant explained: “*Even though you’re sitting in the middle of the forest [in IVR-N], it doesn’t feel as if you’re there. It doesn’t feel real, like in nature, where you’re more enveloped in some way… You’re more affected, all your senses.*” Some participants expressed reservations about IVR-N when it did not evoke the same sense of tranquility they experienced outdoors. In such cases, real nature was clearly preferred: “*When I didn’t get that feeling of tranquility that I get when I sit outside for real, I felt that it just doesn’t work for me. Then it’s better to go sit down in the forest.*” Nevertheless, IVR-N was widely regarded as a valuable complement under specific circumstances. Participants described preferring IVR-N when outdoor conditions were less inviting, such as during poor weather or limited daylight in winter. IVR-N was also seen as particularly beneficial when access to real nature was restricted due to illness, mobility limitations, or other constraints. As one participant noted: “*If I can go outside, that is what I choose. But if I am physically disabled, then experiencing nature on film is wonderful.*”

## 4. Discussion

This study explores experiences of using immersive virtual reality with natural environments as a potential therapeutic support to enhance perceived wellbeing and reduce perceived stress among people with T2D. Participants described IVR-N experiences as providing a sense of tranquility, safety, and mental withdrawal from everyday demands. Experiences were often shaped by familiarity and personal memories connected to the depicted environments. This indicates that the perceived value of IVR-N may be less related to technological immersion per se and more to its capacity to provide accessible conditions for psychological restoration. In the following discussion, we use attention restoration theory as a point of reference to interpret key aspects of the findings, situate the central themes within previous research on IVR-N and nature-based interventions, and discuss potential clinical implications and methodological considerations.

### 4.1. Relation to Restorative Theory

Attention restoration [36] offers a conceptual framework for interpreting the reported experiences, proposing that exposure to natural environments supports recovery from mental fatigue and facilitates cognitive restoration. Similarly, improvements in cognitive functioning and sleep have been documented when being exposed to real-world natural settings, as highlighted in a narrative review by Jimenez et al. [7]. In this context, IVR-N may be understood as enabling key components of restoration even when the physical environment is simulated rather than directly experienced.

### 4.2. Familiarity, Memory, and Personal Meaning

A central aspect of participants’ experiences was the way in which engagement with virtual natural environments intertwined with personal memories and prior life experiences, shaping how the environments were perceived and valued. Huang et al. [34] similarly reported that the IVR brought a symbolic value by precious memories that were awakened by the IVR, the participants returned to places and times from childhood or family gatherings and other meaningful experiences. As described above, in the ART by Kaplan [36], soft fascination provides the possibility to enjoy nature and achieve psychological restoration. For an environment to be restorative, several components are essential, one of which is compatibility. Compatibility refers to the degree to which an environment aligns with an individual’s goals or needs—what one wishes to do or accomplish within that setting. In our study, the goal was to achieve calmness and relaxation. Our findings suggest that positive recollections actively shaped participants’ selection and experience of environments in IVR-N. The role of experiencing familiarity has been reported by Healy et al. [32], who found that familiar content was preferred, especially environments that reminded them of past experiences in real life. Furthermore, familiar environments made the experience more real and enhanced the experience of being in another place [32]. Huang et al. [34] reported that when entering another world, the participant felt separated from frustration, pain, and the depressed situation, and felt relaxed and comfortable. This is in line with the findings in our study, where the participants described being released from everyday demands and worries, which were replaced by moments of freedom and solitude and feelings of calm, relaxation, peace, and security. Taken together, this suggests that familiarity and personal meaning may function as central mechanisms through which restorative effects are achieved in IVR-N, rather than merely contextual influences.

Beyond the immediate experience of IVR-N, several participants described an awakened interest in engaging with real natural environments, suggesting a possible influence on human–nature connectedness. Participants described the desire to spend more time outdoors and to engage with the forest in a different, more mindful way, following their IVR-N experiences. Leopold [40] is often cited as an early ecologist who emphasized the importance of reconnecting humans with nature. Human–nature connectedness refers to the psychological bond with nature, the sense of perceiving oneself as part of the natural world [41]. In a systematic review of meta-analyses, ref. [41] concluded that physical contact with nature enhances psychological connectedness to nature. Both physical and psychological connectedness were positively associated with mental and physical health outcomes [41]. In a meta-synthesis [42], Wood et al. examined which activities most effectively increased human–nature connectedness. Their findings showed that mindfulness-based practices, such as forest bathing and meditation, resulted in the greatest improvements in connectedness [42]. Taken together, these findings suggest that experiences of nature through IVR-N may stimulate reflection and motivation related to real-world nature engagement, with potential implications for psychological wellbeing. This extends previous findings by suggesting that IVR-N may not only replicate aspects of nature exposure but also stimulate engagement with real-world nature through reflective and motivational processes.

### 4.3. IVR-N as a Complement to, Not a Replacement for, Nature

While participants generally experienced IVR-N as calming and acceptable, it was consistently described as a complement to, rather than a replacement for, real-world nature experiences. The immersive environment created a sense of being in nature but also evoked a feeling of being an observer rather than becoming part of nature that could be experienced in real outdoor settings. One possible explanation, expressed by participants, was that only two senses were engaged. In IVR-N, tactile and olfactory perception were absent, which diminished the sense of truly being in nature. It was suggested that IVR-N could serve as a complement for individuals with limited access to real nature. This is in line with Mattila et al. [43] who suggest that VR technology can be effective when access to restorative real nature is limited. Browning et al. [25] evaluated the effects on emotional wellbeing by comparing outdoor and IVR-N experiences among healthy students. They found that both outdoor and IVR-N exposure improved wellbeing compared to an indoor environment without nature. However, while IVR-N reduced negative affect, positive affect remained unchanged, in contrast to outdoor nature, which also increased positive affect. The authors suggested that participants may have become bored or disconnected in IVR-N, which hindered an increase in positive affect. Overall, IVR-N had a more positive effect than an indoor environment but was less effective than real nature [25]. This supports our findings that spending time in real nature may be preferable for enhancing wellbeing, while IVR-N may primarily serve individuals with limited access to natural environments. This distinction highlights that the role of IVR-N may be primarily supportive and context-dependent, rather than substitutive, aligning its use with situations where access to real nature is constrained.

### 4.4. Clinical Implications

The findings point to several potential clinical implications regarding how immersive virtual nature experiences may be used to support wellbeing among people with T2D. Using the IVR-N felt like being transcended from reality into another world. In this virtual environment, everyday demands and worries faded. The sensation of entering another world when spending time in IVR has been reported previously in a meta-synthesis of qualitative studies [34]. However, the study by Huang et al. [34] was from a palliative care context and the VR interventions varied in content. Despite these differences, the experience of being in another world appears to be consistent across contexts. Our findings also confirm Helay et al. [32], who suggested that experiences delivered through IVR may elicit effects comparable to those associated with direct exposure to physical environments. The importance of realism is stressed to be essential for affective response [44]. This suggests that personal preferences play a critical role in determining which natural environments produce the most beneficial outcomes. Consequently, it is reasonable to propose that providing a diverse array of natural environments—particularly those with which users can personally identify—is essential for maximizing restorative potential. These observations suggest that tailoring IVR-N content to individual preferences may be essential for achieving restorative outcomes, highlighting the importance of flexibility rather than standardization in clinical applications.

### 4.5. Methodological Considerations

Although participants described numerous positive experiences of IVR-N, they also reported practical and experiential challenges that may limit the acceptability or effectiveness in some contexts. Complaints about headset functionality were rare; however, when technical issues occurred, they caused noticeable frustration. Some participants describe the headset as heavy and uncomfortable to wear. Additional concerns included the weight of the headset and rapid battery depletion. These findings are consistent with previous research [34]. If headsets are to be used in medical contexts involving frail individuals with declining health, it is essential to consider both the weight of the device and its overall comfort. Since the beginning of our study, it is reasonable to assume that the equipment has been refined, given the rapid pace of technological development.

Our findings also showed that the same environment was calming for one participant but annoying for another, and evoked irritation. A third barrier identified in the findings was the difficulty some participants experienced in establishing a routine for engaging with nature through IVR-N. Some participants perceived the serene environments as monotonous and were drawn to more stimulating activities. This suggests that the intervention may not be universally appealing. Furthermore, it is important to communicate the purpose of the serene environment beforehand, to prepare and encourage users to remain engaged, and to help them overcome any initial restlessness before achieving a sense of tranquility. However, there may also be underlying factors that warrant further reflection and targeted strategies. In everyday life, where the pace is high, it can be challenging to unwind; individuals may need to overcome initial restlessness by practice before achieving a state of tranquility. Feelings of monotony may also be influenced by expectations. When viewing nature films or other recorded material, people often anticipate entertainment; if little occurs, boredom may ensue. Thus, it may be necessary to endure and become accustomed to the tranquil environment before the restorative qualities can be fully experienced. The importance of habituating before gaining positive effects from spending time in nature has been reported earlier. Sonntag et al. [17] found that people with severe exhaustion disorder who participated in forest-based rehabilitation initially felt frustrated. At the beginning, when spending time in the forest doing nothing, feelings of distress and anxiety emerged. However, their mood improved during the rehabilitation period, and they eventually found peace of mind [17]. This variability indicates that user experience is likely to be a key determinant of engagement, suggesting that both individual expectations and intervention design influence the perceived effectiveness of IVR-N.

#### Limitations

As participation in the study was voluntary, those who agreed to take part may have had a particular interest in using digital technologies. That means that a potential novelty effect cannot be ruled out and may influence the transferability of the findings. At the same time, participants expressed a general preference for outdoor nature over IVR, yet suggested that IVR could be useful when access to natural environments is limited.

Seventeen participants took part in the interviews, generating rich and varied data. To enhance dependability, five authors participated in the analysis. The authors’ varying levels of experience with the health effects of natural environments were considered a strength, as this may reduce the influence of preunderstanding on the interpretations. Interpretations, categorization, and theme development were discussed collaboratively to verify and assess the stability of the interpretation until consensus was reached. Participant quotations are included to support credibility. As Krippendorff [45] notes, texts allow multiple interpretations; however, the findings presented reflect what the authors consider the most plausible meanings. The context and participants are described in way to allow readers to assess the transferability of the findings to other settings and groups.

## 5. Conclusions

To our knowledge, this is the first qualitative study to explore experiences of serene natural environments delivered through IVR-N with the explicit aim of stress reduction and enhanced wellbeing in patients with T2D taking part in a multimodal educational program for lifestyle changes.

In conclusion, the findings indicate that spending time in IVR-N seems to be a promising tool that may be supportive for emotional wellbeing and stress reduction, with effects that participants described as lasting beyond the individual sessions. The study also highlights the importance of individual preferences, as the perceived benefits varied depending on how well the virtual natural environment resonated with the individual. Familiarity with the environment appeared to be one factor influencing these experiences. Providing access to a range of different natural environments may therefore be relevant when considering how IVR-based nature experiences can be adapted to diverse user needs.

While IVR-N may not replace actual nature, it can serve as an alternative for individuals with limited access to natural environments. Using the headset also appeared to enhance human–nature connectedness and foster a desire to spend more time in real natural environments to gain strength and recovery.

## Figures and Tables

**Table 1:** Experiences of spending time in nature using IVR.

Themes	Subthemes	Illustrative Content
Finding tranquility and inspiration while transcending time and space	A1 Feeling a peaceful retreat from the demands of everyday life	Feeling calm, being in one’s own bubble, easy access, sense of safety
	A2 Being connected to the past and the present	Memories evoked, familiarity shaping perception
	A3 Feeling a lingering sense of harmony and presence	Lasting calmness, improved focus, better sleep
	A4 Being inspired to engage with nature in new ways	Desire to go outdoors, more mindful presence
	A5 Being hampered to find tranquility	Technical issues, preferences, motivation barriers
B. Perceiving nature in IVR as a surrogate for natural environments	B1 Perceiving nature in IVR-N as comparable to real nature	Perceived realism, sense of presence, familiarity
	B2 Perceiving nature in IVR-N as a complement but not a replacement for real nature	Lack of multisensory engagement, real nature preferred, IVR as complement

## Data Availability

The data sets used and/or analysed during the current study are available from the corresponding author on reasonable request.
